# Chlorophyll-a Retrieval in Turbid Inland Waters Using BC-1A Multispectral Observations: A Case Study of Taihu Lake

**DOI:** 10.3390/s26082535

**Published:** 2026-04-20

**Authors:** Wen Jiang, Qiyun Guo, Chen Cao, Shijie Liu

**Affiliations:** 1 Hangzhou Institute for Advanced Study, University of Chinese Academy of Sciences, Hangzhou 310024, China; jiangwen23@mails.ucas.edu.cn (W.J.); guoqiyun24@mails.ucas.edu.cn (Q.G.); caochen@ucas.ac.cn (C.C.); 2 University of Chinese Academy of Sciences, Beijing 100049, China

**Keywords:** BC-1A, chlorophyll-a, random forest, PCA, remote sensing

## Abstract

Turbid Class II inland waters such as Taihu Lake exhibit a “spectral uplift” effect driven by suspended particulate matter (SPM) scattering and colored dissolved organic matter (CDOM) absorption, which can obscure chlorophyll-a (Chl-a) signals in the visible–red-edge region and challenge retrieval under small-sample, collinear feature settings. Using multispectral observations from the BC-1A satellite (carrying the Lightweight Hyperspectral Remote Sensing Imager, LHRSI) and synchronous satellite–ground in situ measurements acquired over Taihu Lake in late autumn, this study proposes Chl-a-oriented PCA–RF (COP-RF), a leakage-safe inversion framework integrating correlation screening, principal component analysis (PCA), and random forest (RF) regression. Candidate band-combination features are generated, and PCA is applied for orthogonal compression to mitigate collinearity before RF learning. A stratified five-fold cross-validation based on Chl-a quantile bins is adopted, with screening, standardization, and PCA fitted only on training folds. COP-RF achieves stable performance under the current dataset ($R^{2}=0.671$, RMSE =1.80μg/L, MAE =1.25μg/L). Spatial inversion shows higher Chl-a near shores and bays and lower values in the lake center, consistent with Sentinel-2 hotspot ranks.

## 1. Introduction

Lakes are fundamental units supporting regional freshwater supply and ecological security, providing essential ecosystem services such as climate regulation, biodiversity maintenance, and the safeguarding of water resources for production and daily life [1]. With rapid industrialization and urbanization across watersheds, the sustained inputs of nutrients (notably nitrogen and phosphorus) have intensified eutrophication worldwide. Taihu Lake, located in the core of the Yangtze River Delta, is a representative shallow lake and a major source of drinking water, while also undertaking multiple functions related to regional ecological regulation and socio-economic development. Driven jointly by external loading and internal release from sediments, Taihu Lake has remained in a long-term eutrophic state; in summer, the concurrence of high temperature, weak hydrodynamics, and abundant nutrients frequently triggers cyanobacterial blooms, leading to pronounced short-term variability and strong spatial heterogeneity in key biogeochemical parameters [2]. Chlorophyll-*a* (Chl-a), a core indicator of phytoplankton biomass, directly reflects eutrophication status and supports bloom-risk early warning; accurate characterization of its spatiotemporal distribution is therefore crucial for assessing aquatic environmental health and diagnosing remediation effectiveness [3].

Although traditional in situ sampling provides high accuracy, it is typically point-based with limited sampling frequency, making it difficult to represent the rapidly evolving spatial patterns of large shallow lakes under wind–wave disturbance and water-mass transport, and to capture the transient onset and decay of blooms. Satellite remote sensing offers wide-area coverage and periodic repeat observations, providing a feasible pathway toward high-frequency, large-coverage water-quality monitoring systems [4]. However, quantitative remote sensing of Chl-a depends on establishing the response relationship between apparent optical properties and the absorption and scattering characteristics of optically active constituents in water. According to the optical complexity framework proposed by Morel and Prieur, natural waters can be broadly classified into Case I and Case II waters [5]. Case I waters are mainly governed by phytoplankton and related substances, whereas Case II waters are jointly influenced by multiple optically active constituents. Taihu Lake is a typical Case II water body, whose optical properties are jointly controlled by phytoplankton, suspended particulates, and colored dissolved organic matter (CDOM) [6]. The absorption and scattering contributions of these constituents are strongly coupled in the visible bands, resulting in overlapping spectral responses and increased inversion ambiguity [7].

Water-color remote sensing is transitioning from reliance on a few large, operational single-satellite platforms to coordinated observation strategies that combine lightweight payloads with high-revisit systems [8]. For Chl-a retrieval in turbid inland waters such as Taihu Lake, observational capability has long been constrained by trade-offs between spatial resolution, spectral configuration, and revisit frequency. Moderate-to-coarse resolution sensors such as MODIS [9] provide high revisit rates and long time series, enabling large-scale trend monitoring, but they are prone to mixed pixels and adjacency effects in regions with fragmented shorelines and strong nearshore gradients, limiting their ability to resolve fine-scale water-color variability. Dedicated ocean-color instruments (e.g., Sentinel-3 OLCI) offer spectral bands better aligned with water-color processes [10], yet their ∼300 m spatial resolution restricts characterization of localized high-value zones and nearshore structures in inland lakes. In contrast, land-oriented multispectral missions (e.g., Sentinel-2 MSI and Landsat-8/9) provide higher spatial resolution (10–30 m), substantially alleviating mixed-pixel effects; consequently, they have been widely applied for inland-water Chl-a retrieval and bloom detection [11].

The rapid development of China’s spaceborne observation capabilities provides new opportunities for refined monitoring of inland waters [12]. In recent years, the Gaofen series [13,14] and commercial constellations such as Zhuhai-1 [15] have continuously improved in spatial resolution, coverage efficiency, and data-access mechanisms, enriching data sources for routine remote-sensing monitoring of lake environments [16]. In this context, the BC-1A satellite is equipped with the Lightweight Hyperspectral Remote Sensing Imager (LHRSI) [17], achieving a synergy of 10 m spatial resolution and a 200 km swath width under lightweight constraints. This capability can simultaneously support wide-area coverage and fine-scale depiction of nearshore structures, providing new data support for detailed monitoring of inland lakes such as Taihu Lake. For water-color applications, LHRSI spans the visible to near-infrared range and includes key bands in the red-to-red-edge transition, which helps emphasize the coupled absorption, fluorescence, and scattering responses of Chl-a and thus enhances the expression of Chl-a-related spectral-shape differences in turbid waters. Meanwhile, techniques such as time-delayed integration (TDI) improve the radiometric signal-to-noise ratio and image quality, forming a data foundation for quantitative inversion and cross-temporal applications under optically complex conditions. Overall, BC-1A offers an observation-capability combination that is better suited to operational inland-water applications by balancing spatial resolution, swath width, and cost, thereby supporting a China-based water-quality remote-sensing monitoring framework and increasing the practical significance of developing and validating robust inversion models for turbid waters.

Methods for quantitatively estimating water-body Chl-a from remote sensing generally fall into three categories: empirical algorithms, semi-analytical models, and machine-learning/deep-learning approaches [17]. Empirical algorithms establish statistical relationships between Chl-a and remote-sensing reflectance (or its combinations) via regression fitting; they are simple and computationally efficient, and have been widely used in water-color studies. Typical forms include band ratios, differences, indices, and spectral-shape parameters, and index systems can be designed for bloom monitoring in eutrophic waters [18]. In recent years, machine-learning methods such as random forest (RF) [19], support vector regression (SVR) [20], and XGBoost [21] have shown greater robustness in complex optical backgrounds due to their nonlinear fitting capacity, although they remain sensitive to sample representativeness and feature redundancy [22,23].

Against this background, this study uses Taihu Lake as the testbed and integrates synchronous satellite–ground observations to evaluate the applicability and stability of BC-1A for quantitative Chl-a retrieval in turbid inland waters. We develop an inversion pipeline centered on a correlation-constrained principal component analysis (PCA) coupled with RF regression, and quantify model accuracy and generalization under a stratified cross-validation protocol. Basin-wide Chl-a mapping is further produced, and the resulting spatial structures are compared with quantile-ranked hotspot patterns derived from the Sentinel-2 Normalized Difference Chlorophyll Index (NDCI) to examine spatial-consistency characteristics. Key parameter settings and implementation details are provided to support the application of BC-1A multispectral data for inland-water-quality remote sensing and to facilitate subsequent multi-temporal validation.

## 2. Data Sources

### 2.1. Study Area

Taihu Lake (30°55′–31°32′ N, 119°52′–120°36′ E) is located at the southern margin of the Yangtze River Delta and is the third-largest freshwater lake in China, with a surface area of approximately 2338 km^2^ [24]. It is a large, shallow, throughflow lake characterized by a flat lakebed and a mean water depth of about 1.9 m. Influenced by the monsoon climate and intensive human activities, summer conditions are associated with elevated water temperature and increased nutrient loading, making large-scale cyanobacterial blooms prone to outbreak [25]. In winter, lower temperatures and enhanced wind–wave disturbance strengthen water-column mixing, resulting in markedly reduced algal biomass and relatively higher water transparency. Taihu Lake exhibits complex hydrodynamic regimes, where wind-driven currents and basin-scale circulations play an important role in shaping the spatiotemporal distribution of water quality, making it a representative eutrophic shallow-lake testbed for related research [26].

### 2.2. Satellite Data

The BC-1A satellite was launched on 17 January 2025 and carries sensors providing continuous narrowband observations spanning the visible–near-infrared (VNIR) and shortwave infrared (SWIR) ranges. In this study, the 10 m VNIR bands were used as model input features, including the center wavelengths at 412, 443, 490, 555, 620, 670, 690, and 740 nm. Among them, 670, 690, and 740 nm are key bands for chlorophyll-a retrieval. Satellite scenes with cloud cover below 10% were selected. A water mask was derived using the normalized difference water index (NDWI) [27], and valid water pixels were further obtained by removing shadow-contaminated areas. The SWIR bands were used only for aerosol constraint in atmospheric correction and were not included as inputs for the subsequent inversion. The key VNIR band settings of BC-1A used in this study are summarized in Table 1.

### 2.3. In Situ Measurement Time and Locations

**Figure 1:**
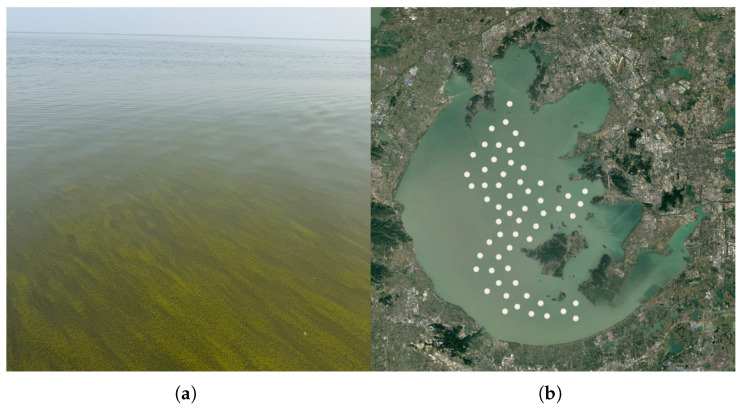
Field campaign over Taihu Lake on 22 November 2025: (**a**) water surface conditions during sampling; (**b**) spatial distribution of 59 valid in situ sampling sites with refined circular markers for clarity.

To establish the correspondence between satellite-derived spectral features and in situ Chl-a measurements, synchronous satellite–ground observations were conducted using a time-matching window of ±4 h around the satellite overpass. A field campaign on 22 November 2025 was carried out over Taihu Lake. The lake surface conditions during the campaign are shown in Figure 1a.

In situ Chl-a concentrations were determined using a portable water-quality analyzer based on the fluorometric principle [28]. During field sampling, surface water at each site was collected and placed in a black container under shaded conditions to minimize interference from ambient stray light. The fluorescence probe of the portable water-quality analyzer was then vertically positioned at a depth of approximately 0.1 m in the black container for measurement. To reduce random measurement error and improve data representativeness, five consecutive measurements were recorded at each site, and their arithmetic mean was taken as the final in situ Chl-a concentration.

Considering potential geolocation uncertainty and small-scale water heterogeneity, the mean reflectance within a 3 × 3 pixel window centered on each sampling site was extracted as the remote-sensing representation of that site. After quality control and stability checks using the coefficient of variation (CV) [29], 59 valid satellite–in situ matchup pairs were retained for model development and validation, as shown in Figure 1b.

## 3. Methods

To evaluate the applicability of BC-1A observations for chlorophyll-a (Chl-a) retrieval in the turbid waters of Taihu Lake, this study established a Chl-a-oriented PCA–RF (COP-RF) framework, in which correlation-constrained principal component analysis (PCA) was combined with random forest (RF) regression. The overall workflow includes four main steps:

1. Data preprocessing: radiometric calibration and atmospheric correction, water-mask generation, and removal of anomalous pixels.
2. Feature construction: generation of candidate predictors from single-band reflectance and band-combination features, including ratios, differences, and normalized differences.
3. Feature compression: Pearson-correlation-based Top-*N* pre-screening followed by PCA-based orthogonal dimensionality reduction to alleviate collinearity.
4. Modeling and validation: $R^{2}$ RF regression modeling within a cross-validation framework, with model performance evaluated using cross-validated predictions and corresponding accuracy metrics such as $R^{2}$ and RMSE. Under the optimal parameter setting, single-scene spatial inversion and comparative analysis are further conducted.

### 3.1. Radiometric Calibration and Atmospheric Correction

**Figure 2:**
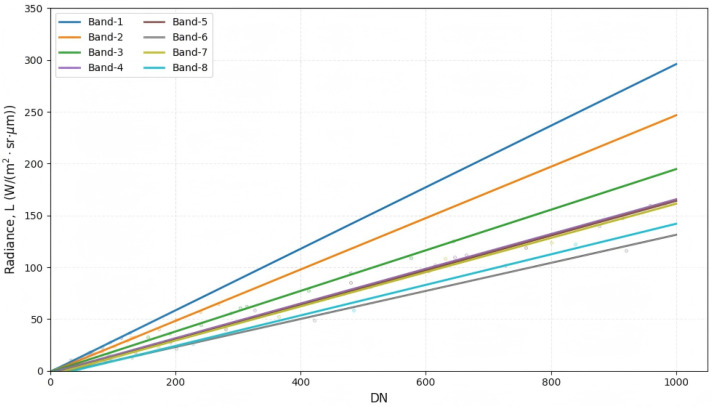
Radiometric calibration curves for the visible–near-infrared (VNIR) bands. *L* denotes at-sensor radiance and DN denotes digital number.

**Figure 3:**
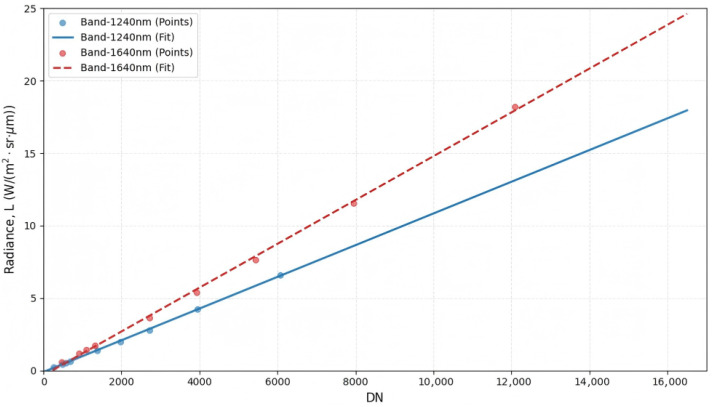
Radiometric calibration curves for the shortwave infrared (SWIR) bands. *L* denotes at-sensor radiance and DN denotes digital number.

To reduce sensor-response inconsistencies and convert raw digital numbers (DNs) into physically meaningful at-sensor radiance (*L*), a linear calibration model was applied:

(1) $$L_{b}=g_{b}\cdot {\mathrm{DN}}_{b}+o_{b},$$

where *b* denotes the band index; $L_{b}$ is the at-sensor radiance (units: W m^−2^ sr^−1^ µm^−1^); $g_{b}$ is the calibration gain (slope); and $o_{b}$ is the offset (bias). Multi-level radiance measurements acquired using an integrating-sphere uniform source were used to estimate $\left(g_{b},o_{b}\right)$ via linear regression. The calibration results show an approximately linear DN–radiance response for both the visible (VNIR) and shortwave infrared (SWIR) bands (Figure 2 and Figure 3). In particular, the non-zero intercept observed in SWIR bands suggests the presence of system dark current, which should be corrected during calibration.

Based on radiometric calibration, atmospheric correction was performed using the FLAASH method [30], which is based on the MODTRAN radiative transfer code. FLAASH was used to estimate path radiance, atmospheric transmittance, and multiple-scattering terms, thereby converting top-of-atmosphere (TOA) signals to surface reflectance. Considering the inland plain setting of Taihu Lake, the rural aerosol model was adopted for atmospheric correction. Because water reflectance is low in the SWIR region, whereas SWIR is relatively sensitive to aerosol scattering, SWIR bands were used to constrain aerosol effects during atmospheric correction, thereby reducing scattering-related bias in the visible bands. To mitigate potential land-to-water radiance contamination in nearshore areas, the built-in adjacency-effect correction in FLAASH was applied during atmospheric correction. SWIR bands were used *only* for aerosol constraint and were not included as inputs for subsequent Chl-*a* inversion.

### 3.2. Water Masking and Removal of Anomalous Pixels

After atmospheric correction, a water mask was generated using NDWI [31] to restrict model training and inference to valid water pixels, and anomalous pixels associated with clouds, cloud shadows, and their neighboring areas were excluded. Based on local trial comparisons and visual interpretation, the NDWI threshold was set to 0.05. Pixels with NDWI>0.05 were classified as valid water pixels, whereas the remaining pixels were regarded as land or shoreline-mixed pixels and removed. Because cyanobacterial bloom regions in Taihu Lake may exhibit abnormally elevated near-infrared (NIR) reflectance, which may reduce the effectiveness of NDWI-based water extraction, the mask was further refined using visual interpretation and cloud-mask products.

For BC-1A, NDWI was defined according to the sensor band configuration as

(2) $$\mathrm{NDWI}=\frac{R_{\mathrm{green}}-R_{\mathrm{NIR}}}{R_{\mathrm{green}}+R_{\mathrm{NIR}}}=\frac{R_{555}-R_{740}}{R_{555}+R_{740}},$$

where $R_{555}$ and $R_{740}$ denote surface reflectance at 555 nm (green) and 740 nm (NIR), respectively.

### 3.3. Feature Construction

Empirical Chl-a retrieval methods commonly use band-combination variables as inputs and approximate the relationship between Chl-a and reflectance via statistical mappings. Considering that turbid waters typically exhibit pronounced variations in overall brightness and a background-scattering-induced uplift of the reflectance spectrum, we summarize the candidate empirical features into two categories of “spectral-contrast” constructions: band ratios and normalized-difference indices. For any two bands *i* and *j*, the two forms are defined as

(3) $$\mathrm{Ratio}\;\left(i,j\right)=\frac{R_{i}}{R_{j}},\;\mathrm{ND}\;\left(i,j\right)=\frac{R_{i}-R_{j}}{R_{i}+R_{j}},$$

where $R_{i}$ and $R_{j}$ denote the surface reflectance at the corresponding band centers. In constructing the candidate feature set, we do not predefine specific band pairs. Instead, all available bands are exhaustively paired to generate ratio and normalized-difference features, and the most effective input forms for Chl-a inversion are subsequently determined through correlation screening and cross-validation.

### 3.4. Pearson Correlation Analysis and Principal Component Analysis

The Pearson correlation coefficient is a statistical measure of linear association between two continuous variables, with values ranging from −1 to 1 [32]. A positive value indicates a positive linear relationship, whereas a negative value indicates a negative linear relationship; the magnitude |r| increases with the strength of linear correlation.

The coefficient is computed as

(4) $$r_{xy}=\frac{\sum_{n=1}^{N}\left(x_{n}-\bar{x}\right)\left(y_{n}-\bar{y}\right)}{\sqrt{\sum_{n=1}^{N}{\left(x_{n}-\bar{x}\right)}^{2}}\sqrt{\sum_{n=1}^{N}{\left(y_{n}-\bar{y}\right)}^{2}}},$$

where $\bar{x}$ and $\bar{y}$ are the sample means of variables *x* and *y*, respectively, and *N* is the sample size.

Principal component analysis (PCA) is a widely used multivariate statistical technique that projects the original high-dimensional variables onto a lower-dimensional space spanned by mutually orthogonal principal components (PCs), thereby enabling dimensionality reduction while reducing redundancy among variables [33]. Let the standardized data matrix be $X\in R^{N\times p}$, where *N* is the number of samples and *p* is the number of original variables. PCA first standardizes X to remove scale effects, then computes the sample covariance matrix and performs eigen-decomposition. The eigenvectors define the directions of PCs, and the eigenvalues quantify the variance explained by each PC. The top *k* PCs are selected in descending order of eigenvalues such that the cumulative explained variance reaches a predefined threshold (e.g., 90% or 95%), yielding a compact and more stable representation for subsequent modeling. Each PC is a linear combination of the original variables, with coefficients given by the corresponding eigenvector.

### 3.5. Random Forest Regression

Random forest (RF) is a supervised learning algorithm under the ensemble-learning paradigm that improves predictive accuracy and stability by aggregating a large number of decorrelated decision trees [19]. Specifically, bootstrap sampling (sampling with replacement) is used to generate multiple training subsets from the original dataset, and a decision tree is trained on each subset. During node splitting, a random subset of features is considered when searching for the optimal split, introducing additional randomness that reduces inter-tree correlation and enhances generalization.

**Figure 4:**
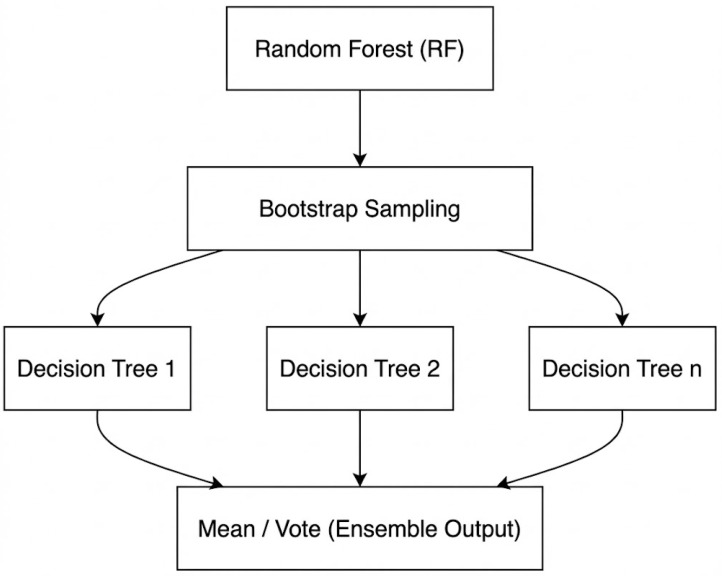
Schematic illustration of the random forest (RF) regression workflow, including bootstrap sampling, randomized feature selection at node splitting, and ensemble averaging of tree predictions.

For regression tasks, RF aggregates the outputs of individual trees by averaging their predictions. Because each tree is trained on different bootstrap samples and randomly selected feature subsets, RF can mitigate overfitting and remains robust to noisy inputs and complex data distributions. Figure 4 illustrates the overall RF workflow: multiple bootstrap subsets are used to train independent trees; each tree recursively splits nodes until the stopping criteria are met; and the final prediction is obtained by averaging the outputs across all trees.

### 3.6. Accuracy Assessment Metrics

Model performance was evaluated using five-fold cross-validation. In each fold, four-fifths of the samples were used for training and the remaining one-fifth for validation. The procedure was repeated five times so that each sample was predicted exactly once by a model that had not been trained on that sample. The out-of-fold predictions from all folds were then aggregated to form a full-sample prediction vector, based on which the coefficient of determination ($R^{2}$), root mean square error (RMSE), and mean absolute error (MAE) were computed as follows:

(5) $$R^{2}=1-\frac{\sum_{n=1}^{N}{\left(y_{n}-{\hat{y}}_{n}\right)}^{2}}{\sum_{n=1}^{N}{\left(y_{n}-\bar{y}\right)}^{2}},$$

(6) $$\mathrm{RMSE}=\sqrt{\frac{1}{N}\sum_{n=1}^{N}{\left(y_{n}-{\hat{y}}_{n}\right)}^{2}},$$

(7) $$\mathrm{MAE}=\frac{1}{N}\sum_{n=1}^{N}\left|y_{n}-{\hat{y}}_{n}\right|,$$

where $y_{n}$ and ${\hat{y}}_{n}$ denote the measured and predicted Chl-a concentrations of the *n*th sample, respectively, $\bar{y}$ is the mean of measured values, and *N* is the total number of matchup samples.

## 4. Results and Discussion

### 4.1. Spectral Characteristics of Taihu Lake Waters

**Figure 5:**
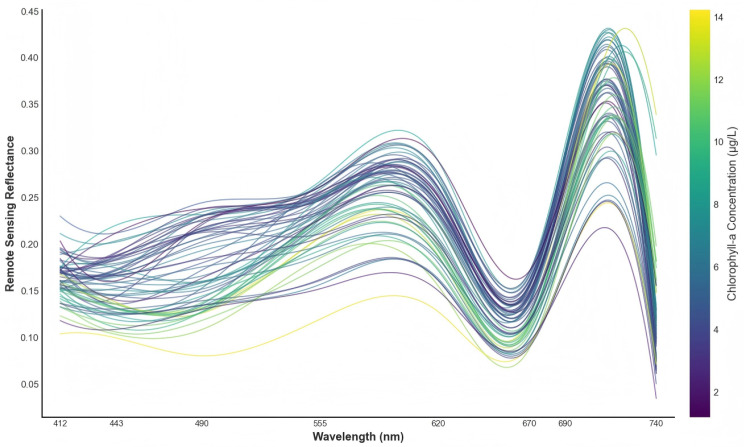
Surface reflectance spectra extracted at the in situ sampling sites over Taihu Lake using BC-1A bands (412–740 nm).

Based on the surface reflectance derived from BC-1A, the bands corresponding to the sampling sites were extracted to form water-leaving spectral curves (Figure 5). Overall, Taihu Lake exhibits spectral-shape characteristics typical of turbid Case II waters across the visible to near-infrared range. Reflectance in the blue region (412–443 nm) is generally low with relatively pronounced fluctuations, which may be attributable to CDOM absorption and residual errors from atmospheric correction. With increasing wavelength, reflectance gradually rises and forms a distinct “green peak” around 555 nm, reflecting enhanced backscattering from suspended particles in the visible domain. It then decreases toward the red region and shows a pronounced trough at 670 nm, corresponding to Chl-a absorption in the red band. In the red-edge region, reflectance rises again around 690 nm and shows a red-edge/near-infrared enhancement response over 690–740 nm. A moderate decline is observed near 740 nm, which may result from the combined effects of increased water absorption and the sensor band setting. Even so, 740 nm still lies at the near-infrared side of the red-edge transition and serves as an effective reference for capturing the contrast between visible-band absorption and near-infrared scattering-related responses. This is also consistent with the feature-screening results in Table 2, where multiple Top-10 features involve 740 nm, such as $R_{620}/R_{740}$, ND(740,490), ND(740,620), ND(740,555), and ND(740,670). It should be noted that LHRSI is a multispectral instrument with a limited number of bands and therefore cannot fully resolve the fine-scale structure of continuous spectra. Despite this limitation, the characteristic combination of a green peak, a red absorption trough, a red-edge rise, and subsequent near-infrared variation shown in Figure 5 still provides key band-level constraints for interpreting Chl-a variability in turbid waters.

### 4.2. Feature Construction and Screening

To better exploit multispectral information and improve Chl-a inversion accuracy, a candidate feature set was constructed using an “original bands + enhanced band combinations” strategy. Specifically, single-band reflectance values were included as baseline predictors, and ratio, difference, and normalized-difference (ND) features were further generated from all possible band pairs. The Pearson correlation coefficient between each candidate feature and in situ Chl-a was then computed; features were ranked by |r|, and the Top-10 were selected as an initial screening result (Table 2).

Table 2 shows that the most strongly correlated predictors are dominated by ratio terms and ND indices combining the 740 nm band with visible bands (e.g., $R_{620}/R_{740}$, ND(740,490), ND(740,620), ND(740,555), and ND(740,670)). This indicates that, under the current data conditions, the contrast between the near-infrared (740 nm) scattering background and visible-band absorption information is more sensitive to Chl-a variability. Correspondingly, several 740 nm based ratio features exhibit negative correlations, reflecting the fact that visible and red-band responses decrease relative to the near-infrared reference as Chl-a-related absorption strengthens. In addition, $R_{690}/R_{490}$ and $R_{690}/R_{620}$ appear in the Top-10 list, suggesting that the red-to-red-edge transition region around 690 nm still contains spectral-shape information relevant to Chl-a. It should be emphasized that Pearson correlation reflects only the strength of linear association and is therefore suitable for quickly discarding weakly related features; it is not equivalent to feature importance in a nonlinear model.

**Figure 6:**
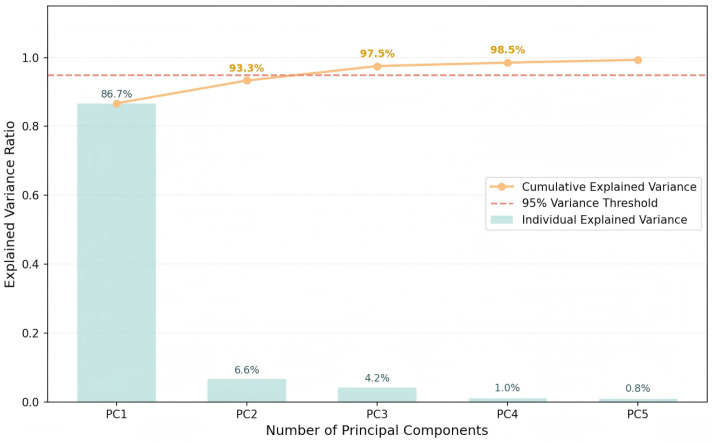
Principal component analysis (PCA) results for the Top-10 screened features, showing the explained variance and cumulative explained variance used to determine the retained components.

Given the strong collinearity among the Top-10 features, principal component analysis (PCA) was applied to the screened feature set to obtain an orthogonal and reduced representation (Figure 6). A cumulative explained-variance threshold of at least 95% was adopted as the truncation criterion. Consequently, the first three principal components were retained for subsequent modeling. The variance contributions of the first, second, and third components are 86.7%, 6.6%, and 4.2%, respectively, yielding a cumulative explained variance of 97.5%. Therefore, the first three components were used as COP-RF inputs to reduce redundancy and mitigate instability induced by multicollinearity.

To further interpret the spectral meaning of the retained principal components, the component coefficients of the Top-10 Pearson-screened features were examined (Table 3). The results show that PC1 is primarily dominated by normalized-difference and ratio features combining 740 nm with visible bands, including ND(740,490), ND(740,555), ND(740,620), $R_{490}/R_{740}$, $R_{555}/R_{740}$, and $R_{620}/R_{740}$. This suggests that the first component mainly reflects the dominant contrast between the near-infrared reference background and visible-band absorption/scattering responses. In contrast, the 690 nm related ratios, especially $R_{690}/R_{490}$ and $R_{690}/R_{620}$, contribute much more strongly to PC2 and PC3, indicating that the red-to-red-edge transition around 690 nm preserves additional spectral-shape variability beyond the dominant PC1 direction. Moreover, ND(740,670) and $R_{670}/R_{740}$ show non-negligible contributions to both PC1 and PC3, implying that the 670–690–740 nm neighborhood jointly reflects coupled variation among red-band absorption, red-edge transition, and near-infrared reference signals. Overall, this coefficient pattern indicates that the retained three-component representation is not only compact but also spectrally interpretable, and may help reduce redundancy associated with dominant background co-variation in the original feature space.

### 4.3. Random Forest Model Development and Inversion Performance

Based on the Top-10 features identified in Section 4.2 and their PCA-based low-dimensional representation, the first three PCs corresponding to a cumulative explained variance of at least 95% were used as inputs to build an RF regression model for Chl-a inversion. By integrating a large ensemble of regression trees, RF can capture nonlinear mappings and typically maintains good robustness and generalization under small-sample and multi-feature settings.

To obtain a reliable evaluation of generalization performance, a stratified five-fold cross-validation scheme based on Chl-a quantile bins was adopted. Within each fold, Pearson screening, standardization, and PCA fitting were performed exclusively on the training set, and the resulting transformations were then applied to the corresponding validation set, thereby avoiding information leakage caused by full-sample preprocessing. After concatenating the out-of-fold predictions from all validation subsets, $R^{2}$, RMSE, and MAE were computed at the full-sample level as accuracy metrics. The model shows stable performance under strict stratified cross-validation: the mean and standard deviation of fold-wise $R^{2}$ are 0.68±0.11, and the aggregated out-of-fold evaluation yields $R^{2}=0.671$, RMSE =1.80μg/L, and MAE =1.25μg/L. The RF hyperparameters were set to $n_{\mathrm{estimators}}=200$, max_depth=10, and random_state=42.

**Figure 7:**
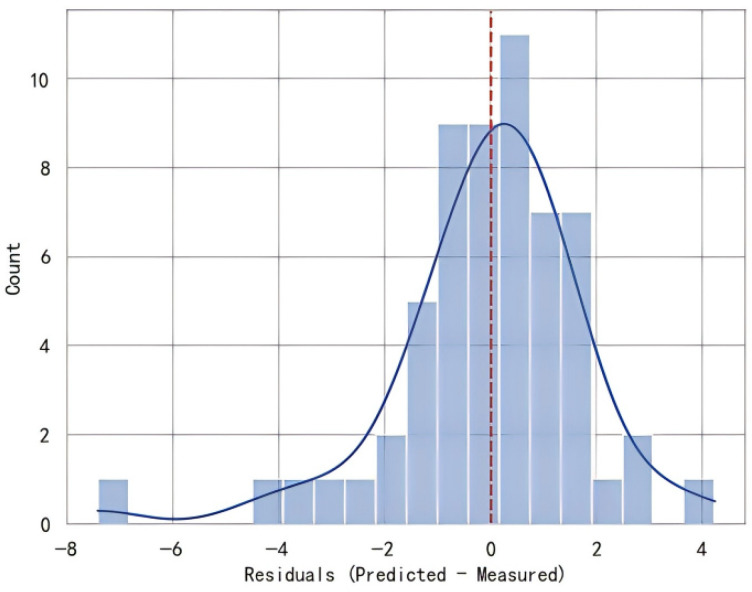
Cross-validated performance of COP-RF: residual distribution derived from stratified five-fold cross-validation.

The corresponding residual distribution is provided in Figure 7. Overall, the residuals are approximately centered around zero, indicating that the proposed model captures Chl-a variability with stable performance under stratified cross-validation.

### 4.4. Model Comparison and Ablation Experiments

For fair comparison, all baseline models and ablation experiments were evaluated under the same stratified five-fold cross-validation protocol based on Chl-a quantile bins. In each fold, Pearson screening, standardization, and PCA fitting were conducted only on the training subset and then applied to the validation subset.

#### 4.4.1. Model Comparison

**Figure 8:**
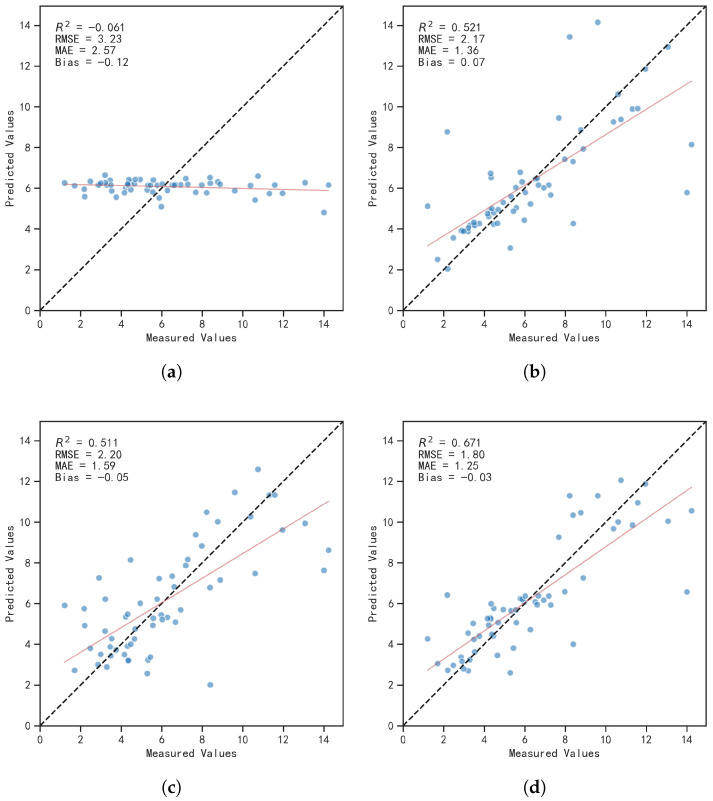
Cross-validation scatter plots and accuracy summaries for different models: (**a**) Ratio (620,670); (**b**) ND (670,740); (**c**) SVR; (**d**) COP-RF.

The aggregated out-of-fold scatter distributions and corresponding accuracy metrics for different models are presented in Figure 8. In each panel, the reported $R^{2}$, RMSE, MAE, and Bias were calculated from the concatenated out-of-fold predictions under the same stratified five-fold cross-validation protocol. Overall, the proposed COP-RF model achieves the best comprehensive performance, with $R^{2}=0.671$, RMSE = 1.80 μg/L, MAE = 1.25 μg/L, and Bias = −0.03. Its scatter points are overall closest to the 1:1 reference line and maintain relatively good agreement across low-, medium-, and high-concentration ranges. For reference, the fold-wise validation $R^{2}$ of COP-RF is 0.68±0.11, which is reported only as a supplementary indicator of cross-fold stability.

In comparison, the support vector regression (SVR) model yields moderate performance ($R^{2}=0.511$). Although it captures part of the nonlinear relationship, it exhibits a clear underestimation at the high-concentration end. Among empirical models, the red–near-infrared normalized-difference index using 670 and 740 nm performs better than other single-variable indices ($R^{2}=0.521$), confirming the physical sensitivity of the contrast between the red absorption trough and the near-infrared reflectance peak for discriminating spectral-shape differences in turbid waters. Although its bias is close to zero, the scatter still shows pronounced heteroscedasticity, indicating that single-variable linear fitting is insufficient to handle nonlinear responses induced by coupled optical constituents. The two-band ratio index Ratio (620,670) has the weakest explanatory power ($R^{2}<0$), showing severe prediction “saturation” and failing to provide effective quantitative discrimination. This result highlights the limitation of simple band-ratio relationships in optically complex turbid waters and further supports the use of multi-feature fusion and ensemble learning in the COP-RF framework. The normalized-difference index (ND(670,740)) can serve as a rapid baseline tool, whereas the proposed COP-RF framework, through multi-feature fusion and ensemble learning, substantially reduces inversion errors and improves predictive consistency, making it more suitable for high-accuracy quantitative Chl-a retrieval in turbid Case II waters such as Taihu Lake.

#### 4.4.2. Ablation Experiments

To quantify the contributions of (i) band-combination feature construction, (ii) Pearson correlation screening, and (iii) PCA-based de-collinearity to inversion performance, five ablation settings (M0–M4) were designed while fixing random forest as the regressor. The evaluation protocol was identical to that described above. As summarized in Table 4, the full pipeline M4 (band-combination features + Pearson Top-10 + PCA with cumulative explained variance ≥95%) achieves the best performance ($R^{2}=0.67$, RMSE =1.81μg/L, MAE =1.25μg/L; fold-wise $R^{2}=0.68\pm 0.11$). The baseline M0, which uses only the original bands, shows substantially lower accuracy ($R^{2}=0.42$). Introducing band-combination features (M1) markedly improves accuracy ($R^{2}=0.62$, RMSE =1.87μg/L) but also results in larger inter-fold variability, suggesting that high-dimensional redundancy can compromise stability. The performance of M2 (PCA applied without prior correlation screening) decreases, indicating that the dominant variance directions may contain substantial background variability weakly related to Chl-a. Using correlation screening alone (M3) already yields relatively high accuracy; however, its inputs still consist mainly of highly correlated ratios and normalized-difference indices, leading to strong collinearity and increased sensitivity to perturbations in the training folds. Building on M3, M4 further applies PCA (≥95%) to orthogonalize and compress the Top-10 correlated features into a small set of principal components, thereby reducing instability induced by redundant correlations while preserving the main information content. The resulting lower overall errors and improved generalization demonstrate that PCA-based de-collinearity is a necessary step for this task.

### 4.5. Spatial Inversion

**Figure 9:**
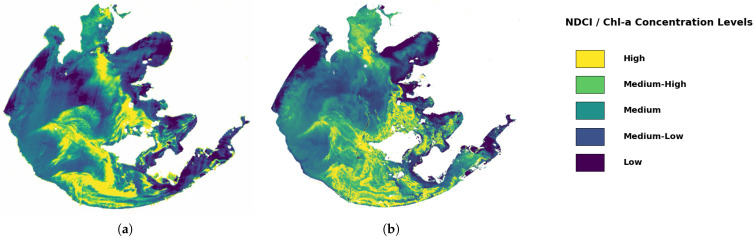
Quantile-ranked spatial patterns over Taihu Lake: (**a**) Chl-a concentration map retrieved from BC-1A on 22 November 2025 and discretized into five quantile classes within the water extent; (**b**) Sentinel-2 NDCI on 23 November 2025 discretized into the same five quantile classes. The common color legend indicates the relative concentration levels. The class intervals are 0–20%, 20–40%, 40–60%, 60–80%, and 80–100% (from low to high).

Figure 9 presents a qualitative quantile-rank comparison of single-scene spatial patterns over Taihu Lake. Using the optimal inversion model and parameter settings described above, Chl-a was mapped from the BC-1A image acquired on 22 November 2025, and pixel-wise results were obtained after water masking and quality control. To emphasize spatial structure while reducing cross-sensor discrepancies in absolute magnitude, we did not compare absolute concentrations directly. Instead, the BC-1A-retrieved Chl-a map (Figure 9a) and NDCI derived from Sentinel-2 on 23 November 2025 (Figure 9b) were independently discretized into five quantile-based classes within their respective water extents (Class 1: 0–20%; Class 2: 20–40%; Class 3: 40–60%; Class 4: 60–80%; Class 5: 80–100%), and a unified color scheme was used to indicate relative intensity.

Because the two images were acquired on adjacent days rather than on the same date, this comparison is intended only as a qualitative assessment of macroscopic spatial consistency rather than a strict validation. The quantile-class distributions show similar large-scale spatial patterns between the two maps. High-class regions (Classes 4–5) are mainly concentrated in nearshore shallow zones, typical bays, and areas adjacent to inflow channels, appearing as banded or patchy clusters. Low-class regions (Classes 1–2) are predominantly distributed over the open central lake and are more spatially continuous, producing an overall gradient pattern of “enhanced nearshore–weakened central lake”. Consistent with the visual comparison, the Spearman correlation between the BC-1A Chl-a quantile classes and the Sentinel-2 NDCI quantile classes over overlapping valid water pixels reached 0.50 (p<0.01), suggesting a statistically significant and moderate positive correspondence in large-scale spatial pattern. Local discrepancies may arise from day-scale wind-field changes that intensify mixing and resuspension, as well as differences between the two sensors in spectral response functions, spatial resolution, signal-to-noise ratio, and residual atmospheric-correction uncertainties. Overall, the quantile-rank comparison suggests that BC-1A can capture the macroscopic spatial heterogeneity of Chl-a in Taihu Lake from a spatial-structure perspective.

## 5. Conclusions

### 5.1. Summary of Main Contributions

This study targets quantitative chlorophyll-a (Chl-a) retrieval in the turbid Case II waters of Taihu Lake. Using multispectral observations from BC-1A and synchronous satellite–ground in situ measurements, we propose a correlation-constrained PCA–random forest regression inversion framework (COP-RF). The framework first applies Pearson correlation screening to identify a feature subspace more strongly associated with Chl-a, then performs PCA within this subspace to obtain an orthogonal, de-collinearized, and compact representation, and finally employs random forest regression to estimate Chl-a. Under a stratified five-fold cross-validation protocol based on Chl-a quantile binning, the proposed method achieves stable performance ($R^{2}=0.671$, RMSE =1.80μg/L, MAE =1.25μg/L), providing a practical reference for applying BC-1A multispectral data to Chl-a retrieval in Taihu Lake under the present late-autumn conditions. Further multi-season validation is needed before extending the framework to broader turbid inland-water applications.

### 5.2. Mechanistic Interpretation and Comparative Analysis

Correlation-based ranking indicates that the Top-10 predictors are dominated by ratio and normalized-difference features combining 740 nm with visible bands, highlighting that the contrast between the near-infrared scattering background and visible-band absorption and scattering information is particularly sensitive to Chl-a variability. In addition, features around 690 nm appear in the Top-10 list, suggesting that the red-to-red-edge transition region still preserves Chl-a-related spectral-shape differences. Although empirical indices such as ND(670,740) are interpretable and computationally efficient, they tend to exhibit heteroscedasticity and bias at high concentrations under optically complex conditions, whereas the proposed COP-RF demonstrates advantages in capturing such nonlinear effects.

This spectral-feature pattern also helps explain the performance differences among the tested methods. Many of the selected predictors combine visible or red bands with the 740 nm band, which acts as a near-infrared reference for expressing the contrast between absorption-sensitive and scattering-sensitive responses. Under these conditions, several 740 nm based ratio features show negative correlations because reflectance in the visible and red bands decreases relative to the near-infrared reference as Chl-a-related absorption strengthens. By contrast, the poor performance of Ratio (620,670), together with its clear prediction saturation, indicates that simple two-band relationships are insufficient to characterize the nonlinear spectral responses of optically complex turbid waters. This result further supports the use of multi-feature fusion and ensemble learning in the COP-RF framework.

The ablation experiments further confirm the necessity of correlation-constrained orthogonal dimensionality reduction. Correlation screening alone can achieve relatively high accuracy; however, the Top-10 features remain highly collinear, which may amplify inter-fold fluctuations under small-sample settings. Introducing PCA on the screened feature set orthogonalizes and compresses the inputs into a few principal components, thereby suppressing instability induced by redundant correlations and improving overall performance and reproducible generalization. In spatial inversion, BC-1A retrievals show a gradient pattern of “higher values near shores and bays and lower values in the lake center”. This macroscopic structure is also in good qualitative agreement with the near-date quantile-ranked hotspot distributions derived from the Sentinel-2 Normalized Difference Chlorophyll Index, supporting the spatial-structure rationality of the BC-1A inversion results. Because the two scenes were acquired on adjacent days, however, this agreement should be understood as qualitative support for large-scale spatial consistency rather than as a strict temporal validation.

### 5.3. Limitations and Future Work

Several limitations should be considered when interpreting the conclusions. First, the number of synchronous satellite–ground matchups is limited and may not sufficiently represent optical states under different seasons and hydrodynamic conditions. In addition, no truly independent spatiotemporal test set was reserved in the present study. Although stratified five-fold cross-validation was adopted and feature screening was conducted within each training fold, the performance assessment still relies on the same set of 59 matchups under an internal resampling framework. Therefore, some risk of optimistic estimation or overfitting may remain under the current small-sample setting, and extrapolation to other times or regions requires further validation. Second, short-wavelength bands in turbid waters are sensitive to residual atmospheric-correction errors, thin clouds, and adjacency effects, and the adopted water-masking and anomalous-pixel removal strategies may introduce systematic uncertainties. Third, the cross-sensor comparison uses quantile discretization to emphasize spatial-structure consistency, which helps avoid misleading conclusions due to incomparable absolute magnitudes, but a strictly harmonized quantitative comparison framework has not yet been established. Fourth, while PCA improves stability, the physical interpretation of principal components requires further analysis using sensor spectral response functions, PCA loading matrices, and sensitivity analyses to enhance mechanistic interpretability.

BC-1A Future work should focus on expanding the BC-1A image archive and increasing the number of synchronous satellite–ground matchups to better capture seasonal and hydrological variability in Taihu Lake. With more data support, an independent cross-temporal or cross-regional test set can be established for more rigorous validation. In addition, future studies may explore stratified modeling, domain-adaptation strategies, and transfer tests across different lake regions to assess the broader applicability of the proposed framework.

## Figures and Tables

**Table 1:** Key band parameters of the BC-1A multispectral sensor used in this study.

Band	Center Wavelength (nm)	Bandwidth (nm)	Spectral Characteristics
B1	412	30	Strong absorption by CDOM and detritus; most sensitive to atmospheric scattering.
B2	443	30	Blue absorption peak of chlorophyll-a (first absorption peak).
B3	490	30	Carotenoid absorption; auxiliary band for pigment-related retrieval.
B4	555	30	Green reflectance peak; baseline reference for turbid-water spectral shape.
B5	620	30	Diagnostic absorption of phycocyanin; key band for cyanobacteria detection.
B6	670	30	Red absorption trough of chlorophyll-a (second absorption peak).
B7	690	25	Onset of red-edge/fluorescence peak; sensitive to high Chl-a concentrations.
B8	740	40	Near-infrared reflectance plateau; dominated by particle scattering with weak pigment absorption.

**Table 2:** Top-10 candidate features ranked by absolute Pearson correlation with in situ Chl-a.

Rank	Selected Feature	Pearson *r*
1	$R_{620}/R_{740}$	−0.7877
2	ND(740,490)	0.7845
3	ND(740,620)	0.7840
4	ND(740,555)	0.7803
5	ND(740,670)	0.7754
6	$R_{555}/R_{740}$	−0.7729
7	$R_{690}/R_{490}$	0.7710
8	$R_{670}/R_{740}$	−0.7706
9	$R_{690}/R_{620}$	0.7615
10	$R_{490}/R_{740}$	−0.7569

**Table 3:** PCA component coefficients of the Top-10 Pearson-screened features retained for COP-RF modeling.

Feature	PC1	PC2	PC3
$R_{620}/R_{740}$	−0.338	0.145	−0.192
ND(740,490)	0.369	−0.116	0.092
ND(740,620)	0.345	−0.189	0.217
ND(740,555)	0.362	0.084	−0.110
ND(740,670)	0.331	0.215	0.340
$R_{555}/R_{740}$	−0.349	−0.093	0.119
$R_{690}/R_{490}$	0.148	0.667	0.445
$R_{670}/R_{740}$	−0.324	−0.202	−0.307
$R_{690}/R_{620}$	0.124	−0.597	0.687
$R_{490}/R_{740}$	−0.355	0.158	−0.058

**Table 4:** Ablation results of the random forest regressor under the same stratified five-fold cross-validation protocol.

ID	Combo	Top-10	PCA	$R^{2}$	RMSE (μg/L)	MAE (μg/L)	Fold $R^{2}$
M0 (Baseline)	✕	✕	✕	0.42	2.12	1.45	0.42 ± 0.12
M1	✓	✕	✕	0.62	1.87	1.51	0.62 ± 0.17
M2	✓	✕	✓	0.54	2.00	1.28	0.55 ± 0.13
M3	✓	✓	✕	0.59	1.89	1.34	0.59 ± 0.13
M4 (Ours)	✓	✓	✓	0.67	1.81	1.25	0.68 ± 0.11

✓: module applied; ✕: module omitted. Combo: band-combination features; Top-10: Pearson |r| Top-10 screening; PCA: PCA-based dimensionality reduction with cumulative explained variance ≥95%.

## Data Availability

The data presented in this study are available on request from the corresponding author. The data are not publicly available due to ongoing project restrictions.
